# The Mystery of Life

**Published:** 1871-05

**Authors:** Jno. M. Woodworth

**Affiliations:** President


CHICAGO

MEDICAL EXAMINER:

N. S. DAVIS, M.D., EDITOR.

F. H. DAVIS, M.D., ASSISTANT-EDITOR.

VOL. XII. MAY, 1871. NO. 5.

### Original Contributions.

### THE MYSTERY OF LIFE.

AN ADDRESS DELIVERED BEFORE THE ALUMNI ASSOCIATION OF THE CHICAGO MEDICAL COLLEGE.

BY JNO. M. WOODWORTH, A.M., M.D., PRESIDENT.

The seeds found in Egyptian tombs, on being planted in the soil of France, grew into waving wheat.

For three thousand years these grains had been without life, yet we cannot say they were *dead;* they were organic seeds, products of life, and had themselves once *lived;* they were developed from cells into seeds, and at this point in their development their vital activity was arrested, because the necessary heat, light, and soil was wanting; but, no sooner were these supplied, than the arrested activity was once more set free—the seed became plants.

Frogs and toads, when subjected to the action of intense cold, may be perfectly *frozen;* yet these animals are not dead. Let heat be cautiously applied, and in a few minutes life is once more in full activity.

The microscopic animals known as Rotifers become, to all appearances, dead, when the water of the moss in which they live is evaporated; and in this state of suspended animation they may remain for years, recovering their energy on the addition of a little water; they may be dried and revived many times in succession; but they are not dead in these cases, they do not decompose.

I have myself dried the horned frogs of the South, until they were brittle, and to all appearances as completely destitute of life as a chip of wood; in this condition I have kept them for months; but, on putting them in a wet place, in a few hours they were full of life.

In all these cases the phenomena we call life is entirely absent; it would *never* manifest itself, unless the peculiar condition necessary for its manifestation was supplied.

The three thousand years would, for the Egyptian grain, stretch to an eternity. The animal organism in this state of suspended animation is not destroyed; it is like a watch that has run down. .

Life is indeed a mystery, whether considered as manifested in plants or animals; with nothing are we more familiar; of nothing do we know less.

When we seek to know the vivifying power first manifested in the germ, we approach the mysteries of the Creative Being. This we may not know until we have solved the equally great mystery, death.

Yet, there are problems concerning the vital principle; its first appearance and locality; its manifestation through the mind; as well as the creative and preserving power carried on by it in our physical organism, of which our mind is unconscious, and over which it has no control.

These questions may well claim our study.

In the popular mind no distinction exists between life, the mind, and the soul of man; all are confused together; and all are thought to compose a single immaterial spirit, which comes at birth and goes at death—let us hope to the source from whence it came.

The vital principle has been compared to air; has been supposed to exist in the blood; in the stomach; in the brain; in the liver; in the diaphragm; and in the heart.

Perhaps the theory that obtains more generally than others, at the present day, is that the soul pervades the whole body alike, with the royal seat of life in the brain.

Let us inquire if this be true? We know that extensive injuries to the brain and spinal cord are not unfrequently borne with comparative impunity. I have seen a man who was shot through the head, the ball cutting off the optic nerves in its course, and yet the man recovered without perceptible injury to his faculties, except the loss of sight. I have scooped out considerable quantities of the substance of the human brain, and yet the persons recovered, and all the phenomena of life went on the same as before.

Life has been kept up in animals after the brain has been removed, by simply supplying artificial respiration.

In fishes, the heart will beat for hours after the brain and spinal cord have been taken away.

A frog has been known to live for six months after the whole brain had been removed. A large turtle knocked the knife out of my hand with its foot when I attempted to remove the shell, two hours after I had severed the head from the body.

Instances might be multiplied, but these will suffice—we must look to some other part of the body to find the seat of life.

The organic or sympathetic nervous system is that part of the living animal which is first formed in embryo.

The sympathetic nerves of the chest and abdomen are fully developed while the brain is still a pulpy mass.

The invertebrates have no brain or spinal cord. If we examine their nervous organization we shall find that it corresponds to the organic nervous system of the higher animals.

In the polyps and echinoderms it is very simple, consisting of a few ganglia which surround the œsophagus; in insects it is more complex, and beside the œsophageal ring, which has been compared to the brain, there exists a ventral chain of ganglia from each of which, nerves are given off to supply the viscera and other organs. There are animals without a head or brain, without a heart or liver, and even without a stomach (unless we can stretch our imagination enough to call a slight depression on the outside of the body, a stomach), and if we exclude animals too small for examination, it may be said of all animals alike, they each possess an organic or sympathetic nervous system. Is it, then, too much to say, that the sympathetic nervous system is the source of all animal life.

The dense network of nerves situated behind the stomach, called the *solar plexus*, which is the root of the sympathetic nervous system, is the first part of our bodies that makes its appearance and takes form in embryo. Around this germ of development, in obedience to the Great First Cause, the house in which we live is built up, and through it, and its connecting nerves, in obedience to a sleepless Providence, our tenement is kept in repair and preserved from danger.

The functions of the sympathetic nervous system (with the exception of generation) are as perfect at birth as at adult age, which cannot be said of the mind and brain.

The silent unconscious process continually going on within our bodies, by which the waste is repaired—sending the right particles to the right place, and excluding the worn out and worthless—the influence which propels the little engine that started within our bodies in the morning of our existence, beating on night and day, whether we sleep or wake, never stopping for repair, but laboring on till life's work is done, is, to my mind, governed by the same influence that teaches the infant to draw nourishment from its mother's breast.

The mind has no part in these instinctive acts, but as they are essentially processes of preservation, may they not be said to originate from the solar plexus (which appears to be the seat of life) in the same sense that the brain is the organ of the mind.

We have seen that hard blows on the head are often received with comparative impunity. Slight blows over the solar plexus give rise to an indescribable feeling of agony, and heavier blows over this organ have frequently caused death.

That there is a certain informing spirit within us, which is not of the mind, all will, I believe, admit. It comes to the least of us, as a voice that will be heard; it warns us of danger; it tells us what we must believe; it frames our sentences; it is the inspiration of the poet and the artist; it is the hidden power of the orator; it lends a sudden gleam of wisdom or eloquence to the dullest of us all, so that we often wonder at ourselves. This same prompter of our waking hours, like a faithful sentinel, stands guard while the mind sleeps.

Who has not noticed, when about to start on a journey early in the morning, by fixing in the mind before going to sleep the hour necessary to awaken, at that very hour they have started into consciousness as if shaken by the hand of a friend.

The weary soldier may sleep while standing at his post, but the muscles necessary to retain his erect position do not relax.

I have often slept seated in the saddle, while my horse travelled on for miles, but when my muscles became fatigued and were about to loose their tension I was apparently awakened by a dream.

You have seen a mother in a railroad car, holding her infant when tired nature had forced sleep upon her, her head will drop upon her breast, but her arms do not sleep, and when the exhausted muscles are about to relax and let the child fall, the loving mother will awaken with a start and gather her babe to her breast.

I have often marvelled at this mysterious guardian, and as often asked myself what part of us it is that remains awake to arouse the sleeper or guard the infant.

Inspiration teaches that the spirit and the understanding are distinct and separate. May not the solar plexus be the instrument of the former in the same way that the brain is the servant of the latter?

That it is the seat of our emotions seems more than probable.

Who has not felt love and joy "that fills the heart"—(we never say fills the head or mind)—and many of us are not strangers to the imprisoned agony of grief that has entered here until the load has become an actual physical pang; so keen that we fain would rend the very garments that lay their burdensome restraint upon our bosoms. Upon this theory it is easy to account for the impulse that clasps a beloved object in a warm embrace, and to understand the peculiar bliss experienced by a happy mother, as soft hands steal about her neck, and she presses her babe close, and yet closer in her circling arms.

As the mind develops in power and strength, certain of the instinctive and the emotional faculties are, to some extent, superceded and controlled by it.

A familiar illustration of the influence of condition and education over the instinctive faculties in the lower animals may be seen in the Canary bird; it cannot build its own nest, nor provide for itself; if turned loose in its own native island it would soon die.

In children, in both sexes, the emotional nature predominates over the intelligence and judgment. The boy in passing to manhood, through the ruggedness and sterness of his training, with many hard knocks thrown in by way of punctuation, looses much of his impressibility, and as a general rule the emotional nature becomes less active as the intellectual and physical increases. This, however, is not true in all cases. The influence of the Christian religion, or some defect or impediment, causing timidity or iregularity of action in the outer world, may preserve and foster the emotional nature in all the freshness of youth.

The gentler sex possess greater warmth of emotion than man, and submit to its influence rather than to logic. Woman is man's superior in the emotional world; his equal in the moral world; and in the laboring his inferior.

She retains the characteristics of her childhood, though in an intensified degree, and is nearer to the point of departure of that development which outlives time and peoples heaven; and if man would reach this higher level, he must retrace his steps and regain something he lost in his youth.

This may appear strange to the executing, commanding, reasoning man; and yet not so strange to those who realize the significance of the law uttered by Christ—"Except ye become as little children ye cannot enter the kingdom of heaven." If what I have stated be true, the much agitated "woman's rights question" would appear in a new light; and, instead of trying to solve the question by the ballot, *man* should seek the higher level, which is the heritage of the *true* woman, by joining to the ruggedness of his character more of submission, love, and faith, which the new birth alone can give.

Dr. Hammond asserts that the brain secretes mind, and the liver and stomach secrete bile and gastric juice, respectively. How vain must be the attempt of Dr. Hammond, or any other doctor, to find the soul by a *post mortem* examination!

We use the words here, mind and soul, as synonymous terms, but strictly speaking the soul comprehends more than the mind; the latter is confined to the rational principle, while the former not only contains this, but the instinctive power also.

The soul contains mind and life also; how intimate their relation with the physical organism.

To find out the action and reaction of the mind and the emotions on the body, and on each other, is the greatest problem of the physician; when it is better understood, and our actions governed accordingly, we shall live happier, purer, healthier lives.

We make a lion of the athlete of the period; we stand in wonder of the spectacle of that giant of German learning, Neander, lying his whole length on the floor among his books, forgetful of time or place, not knowing whether he is on the earth or in the moon, led like a child by his sister to his lecture room when the lecture hour came, and led away home again when it was over; in one, the emotional nature runs riot in wild passion; in the other, love, hope, and pity have withered away and become as useless as a palsied limb; they are types of *deformed* men, and both to be pitied alike.

Some men hold the mind in paramount esteem; others the body. Here, brain is had in honor; there, muscle and sinew. But the men who would fill up the measure of life must have strong limbs and thoughtful minds.

The culture of the mind is not necessarily made at the expense of the body; but, on the contrary, mental application is a powerful remedy in diseases both of body and mind.

We hear grave charges made against mind labor, but the evil does not result so much from what is done as from what is neglected.

The school is very apt to get the credit of spoiling constitutions which, in point of fact, it only finds weak and leaves as it found them.

When nature makes a great man, she presents him with a good constitution. "But," you ask, "has that lean man, with a face like parchment, and not much flesh on his bones, a constitution?" Yes, he has, he has a working constitution, and a better one than you, with your ruddy face and well-rounded form.

You may be the "very picture of health," and have only a sporting constitution, not a *working* one. You may get on very well with exercise in the open air, and no strain on your brain, but our lean friend can stand confinemcnt and long hours of mental labor without fatigue, from which you would emerge pale and "used up."

Nature may not have made a handsome man of our lean friend, but what does that signify? She made him a strong one.

The mind must be gradually inured to labor to be used with ease and profit. Let a man walk thirty miles in one day without training, and he will be painfully impressed with the fact that the body has laws which cannot be violated with impunity. The brain tissues cannot be expected to be *more* tolerant of such liberties than the muscles.

Many a boy, the victim of hereditary influences, gives promise of future greatness; but, like phosphorescent insects, their brilliance lasts but a little while, and is at its height when on the point of being extinguished forever. If, by any means their downward tendency be stopped, it is extremely rare that the autumn of life fulfils the promise of its spring.

Fatal disease is frequently induced in children by forced mental exertion, and, where death does not follow, the moral perception is apt to be obscured, and the sensitive child becomes a man of hardened vice, or of ungovernable self-will.

Examples are not wanting to show that devotion to intellectual pursuits and to studies, even of severe and unremitting character, is not incompatible with extreme longevity, terminated by a serene and unclouded sunset. Where one man is injured by mental application, two are injured by athletic sports, and hundreds by intemperance and other vices to which idleness leads.

Wearying anxiety, and the depressing passions and emotions generally, are so many disturbing elements in the machinery of life. But idleness is the greatest enemy of mankind, and in the American grammar it belongs to the feminine gender!

Fellow-laborers, my task is done. We have met for the first time in the new temple of our Alma Mater, to exchange our annual greeting. Some may have expected words of congratulation upon this particular occasion, when each of us might be pardoned for feeling honored by the new and well-fitting dress in which our benign mother greets us. But I take it, that we should come here to contribute some of the proceeds of our stewardship, rather than borrow from the old lady.

The first graduates from this institution went directly from her unpretending lecture-rooms to pitch their tents on the field of battle.

Those who go out now will not be tried by the horrors of war, nor share the inspiration of its pomp and circumstance; yet the battle of life and death is often fought as really in chambers or in an office as it is on the field,—but remember, the victory is to the strong.

If I could utter words that would reach those who are to come after us, I would say: Fear not to do manfully the work for which your gifts qualify you; but do it as one who must give account both of body and soul. Work hard while it is day, the night cometh soon enough—do not hasten it.

Use your faculties to their full capacity, but do not abuse them.

The body has its claims, it is a good servant; treat it well and it will do your work; it knows its own business, do not attempt to force it; listen kindly and patiently to its hints; attend to its wants and requirements, and you will be rewarded. But task it, and pine it, and suffocate it, make it a slave instead of a servant; it may not complain much, but, like the weary camel in the desert, it will lie down and die.

